# Clinical Characteristics of Target Organ Damage in Primary Aldosteronism with or without Metabolic Syndrome

**DOI:** 10.1155/2022/8932133

**Published:** 2022-09-07

**Authors:** Xiaona Bu, Fang Sun, Hexuan Zhang, Xiaoli Liu, Zhigang Zhao, Hongbo He, Yingsha Li, Zhencheng Yan, Zhiming Zhu

**Affiliations:** Center for Hypertension and Metabolic Diseases, Department of Hypertension and Endocrinology, Daping Hospital, Army Medical University of PLA, Chongqing Institute of Hypertension, Chongqing 400042, China

## Abstract

The aim of this study is to investigate the prevalence of metabolic disorders in patients with primary aldosteronism (PA) and target organ damage (TOD) in different subtypes of patients with PA with or without metabolic syndrome (MS). Patients with PA were screened out from those with secondary hypertension and then subtyped via adrenal venous sampling (AVS). Baseline clinical characteristics (blood pressure, blood glucose, abdominal circumference, and lipid profile) were collected for the diagnosis of MS. Organ damage was evaluated according to cardiac and carotid ultrasound and urine microalbumin measurements. In all 261 patients with PA, 113 patients had concomitant MS and experienced more severe cardiac hypertrophy and increased intima-media thickness (IMT). The incidence of MS and diabetes mellitus (DM) had no statistic difference between the two groups, moreover, the rates of TOD were not different except microalbuminuria. However, metabolic disorders caused more remarkable TOD in PA patients with unilateral hypersecretion. It showed that cardiac hypertrophy was associated with obesity while microalbuminuria was related to plasma aldosterone concentration (PAC) in PA patients. In this retrospective study, our findings suggest that the effect of metabolic disorders on organ damage is more remarkable in patients with unilateral PA.

## 1. Introduction

Primary aldosteronism (PA) is a common form of endocrine hypertension characterized by autonomous aldosterone overproduction without negative feedback regulation of upstream hormones, usually accompanied by hypertension and hypokalemia [1–3]. Patients with PA often have multiple metabolic disorders, including insulin resistance, obesity, and dyslipidemia [4, 5]. Patients with PA reportedly have an increased incidence of metabolic syndrome (MS) compared with patients with essential hypertension (EH) [6–9], while another study had contradictory findings [10]. A positive correlation between PAC and obesity and metabolism has also been reported [11, 12]. In addition, aldosterone excess has a deleterious effect on cardiovascular tissues independent of blood pressure [13]. Some studies reported that patients with PA have a higher risk of cardiovascular and cerebrovascular events than patients with EH [8], but not in others. However, the impact of MS on target organ damage in patients with PA is poorly elucidated.

PA has two main clinical subtypes: idiopathic hyperaldosteronism (IHA) and aldosterone-producing adenoma (APA). Laparoscopic adrenalectomy is the principal treatment for patients with APA or unilateral adrenal hyperplasia, whereas mineralocorticoid receptor (MR) antagonists are mainly recommended for patients with IHA. Our previous study showed catheter-based adrenal ablation can reduce blood pressure in patients with PA and also effectively reduce medication burden and aldosterone levels [14]. A study showed that patients with IHA have a higher occurrence of metabolic disorders [15]. Recently, a multicenter cohort study reported that prediabetes is more prevalent in bilateral PA than in unilateral PA [16]. However, few studies have discussed how metabolic disorders contribute to target organ damage (TOD) in patients with different clinical subtypes of PA.

To date, previous studies have concluded that CT scans have a limited role in subtype classification, especially in nonfunctioning adenomas or smaller lesions. Thus, adrenal venous sampling (AVS) is used as the gold standard for aldosterone lateralization [17]. In our previous study, we also found a 50% inconsistency rate between CT and AVS among 32 cases [18]. Therefore, the aim of our present study was to examine the prevalence of metabolic disorders in different subtypes of patients with PA diagnosed via AVS, as well as to determine whether the presence of MS aggravates the severity of organ damage.

## 2. Materials and Methods

### 2.1. Patients

All patients were recruited from the screening of hypertensive individuals who were hospitalized in the Department of Hypertension and Endocrinology of Daping Hospital from January 2013 to January 2020. Before screening, all of them had stopped antihypertensive drugs (i.e., *β*-receptor blocker, angiotensin-converting enzyme inhibitor, or angiotensin II receptor antagonist) for more than 2 weeks, aldosterone receptor antagonist for more than 6 weeks, and other diuretics for more than 4 weeks. Either or both nondihydropyridine calcium channel blockers and *α* receptor blockers were provided to patients with uncontrolled hypertension. Hypokalemia patients (≤3.5 mmol/L) were treated with potassium supplementation. All participants were instructed to maintain their usual diet.

We excluded patients with other causes of secondary hypertension, including renal hypertension, renovascular hypertension, and adrenal hypertension (i.e., pheochromocytoma and Cushing syndrome). Patients who recently experienced stress states, such as infection, trauma, and surgery, as well as those with a history of severe cardiac disease, hepatorenal insufficiency, and cardiovascular disease, were also excluded. No patients were given glucocorticoids, licorice preparations, or contraceptives. This study complied with the tenets of the Declaration of Helsinki and was approved by the ethics committee of Daping Hospital, Army Medical University of PLA. Written informed consent was obtained from all patients.

### 2.2. Diagnosis and Classification of PA

Among the initial number of participants, those with an increased aldosterone-to-renin ratio (ARR) and positive saline infusion confirmatory testing underwent AVS for subtype classification of PA. Those with ARR > 20 ng/dL·ng/mL/h were considered to have a positive screening result according to the reference scope of the laboratory [3]. Patients with PAC > 10 ng/dL after intravenous saline infusion were considered to have PA, while those with PAC < 5 ng/dL were excluded. In addition, patients with PAC 5–10 ng/dL were diagnosed on the basis of consistent clinical and biochemical parameters, including serum potassium concentration and the results of an adrenal computed tomography scan. Having a high secretion of aldosterone that cannot be inhibited on the confirmatory test would lead to a diagnosis of PA [19].

AVS was performed in all patients without adrenocorticotropic hormone simulation, and this was performed as described in our previous study [18]. Sampling was regarded as successful when the selectivity index (SI, defined as cortisol in the adrenal vein/cortisol) was ≥2. Unilateral aldosterone overproduction was considered as having a lateralization index (the cortisol-corrected ratio) of ≥2 based on expert consensus [20].

### 2.3. Definition of MS

According to the Chinese Guidelines for the prevention and treatment of type 2 diabetes mellitus (DM) in 2017 [21], MS was diagnosed in patients who had at least three of the following: (a) abdominal obesity: waist circumference (WC) ≥ 90 cm in men and ≥85 cm in women; (b) hypertension: systolic blood pressure ≥ 130 mmHg or diastolic blood pressure ≥ 85 mmHg or previously diagnosed with or treated for hypertension or both; (c) hyperglycemia: fasting plasma glucose (FPG) ≥ 6.1 mmol/L (110 mg/dL), 2 − hour postprandial blood glucose ≥ 7.8 mmol/L (140 mg/dL), or previously diagnosed with DM; and (d) dyslipidemia: triglyceride (TG) ≥ 1.7 mmol/L (150 mg/dL) or high − density lipoprotein cholesterol (HDL − c) < 1.04 mmol/L (40 mg/dL) or both.

### 2.4. Laboratory Assessment

The following basic clinical data were collected: name, sex, age, body mass index (BMI), waist circumstance (WC), duration of hypertension, and office blood pressure. The following laboratory measurements were obtained from patients' fasting venous blood samples: total cholesterol, TG, HDL-c and low-density lipoprotein cholesterol (LDL-c), sodium (Na), potassium (K), hepatic enzyme, uric acid (UA) levels, creatinine (Cr), FPG, and glycated hemoglobin A1c (HbA1c). Plasma renin activity (PRA) and PAC were also measured in the orthostatic and recumbent positions via radioimmunoassay and chemilumininescentimmuno assay (Italian Diasorin, LIAISON Aldosterone, and Direct Renin Concentration Kit). All biochemical tests were carried out by a professional technician in our laboratory.

### 2.5. Evaluation of Organ Damage

Hypertensive heart disease was mainly characterized by the presence of left ventricular hypertrophy (LVH). Coronary artery disease was diagnosed if patients had myocardial ischemia by electrocardiography or myocardial perfusion scintigraphy or coronary stenosis by coronary angiography. Left atrial diameters (LAD), left ventricular diameters (LVDd), interventricular septum thickness (IVST), and left ventricular posterior wall thickness (LVPW) at the end diastolic and end systolic phases were calculated via two-dimensional guided M-type mapping. Left ventricular mass (LVM) was calculated using the Devereux Formula [22] and normalized by body surface area (BSA). The reference formula is as follows: LVMI = LVM/BSA. According to the European hypertension guidelines [23], LVH was defined as LVMI > 115 g/m^2^ in males and >95 g/m^2^ in females.

Renal dysfunction was defined as having either or both estimated glomerular filtration (eGFR) of <60 ml/min per 1.73 m^2^ using the CKD-EPI equation and with microalbuminuria, defined as having a 24 h urinary albumin to creatinine ratio (UACR) ≥ 30 mg/g. The 24 h urinary albumin was measured via nephelometry (detection limit, 0.1 mg/dL, American Backman Company Kit).

Echocardiography and determination of the carotid intima thickness were performed by the same investigators. Carotid intimal thickening was diagnosed as having a carotid IMT > 0.9 mm [24]. Cerebrovascular accidents included ischemic and hemorrhagic stroke, either previously or newly diagnosed.

### 2.6. Statistical Analysis

Statistical analyses were performed using the Statistical Package for the Social Sciences (SPSS version 18.0; SPSS, Chicago, IL) software. We apply Shapiro-Wilk test to assess the normality. Normally distributed data are presented as the mean and the standard deviation (SD). Nonnormally distributed counting data are represented as the median (M) and 25^th^ and 75^th^ percentiles and were analyzed using the Mann–Whitney *U* test. Univariate analyses between two groups were performed using *t* tests. Analysis of categorical data was assessed using the *χ*^2^ test. The strength of the correlation between variables was tested by Spearman's correlation. Logistic regression analysis was performed to determine the effect of variables on organ damage. A *P* value < 0.05 was considered statistically significant.

## 3. Results

### 3.1. Clinical Characteristics of Patients with PA with or without MS

A total of 261 patients with PA were included in this study, with 113 (43%) having MS. The clinical characteristics of these patients are summarized in Table 1. Overall, PA patients with MS were older (52 ± 10 vs. 47 ± 11) and more commonly male (58.4% vs. 38.4%) than PA patients without MS. PA patients with MS also had higher BMI, WC, TG, and UA levels but lower HDL-c levels than PA patients without MS. PAC, PRA, and serum K were not different between the two groups.

### 3.2. Target Organ Damage and Cardiorenal Complications in Patients with PA with or without MS

In comparison to patients with non-MS PA, patients with MS had markedly greater LAD, LVDd, IVST, and LVPW, along with significantly increased IMT, higher plasma Creatinine level, and lower eGFR. According to the *χ*^2^ test, patients with PA with MS had higher incidences of hypertensive heart disease (29.7% vs. 53.1%, *P* < 0.001) and cerebrovascular disease (22.9% vs. 44.2%, *P* < 0.001) than those without MS. There was no difference in the incidence of coronary heart disease and chronic renal disease between the two groups (Table 2). We conducted logistic regression analysis to determine the factors affecting organ damage (Table 3). PAC, TG, and obesity were significantly associated with cardiac hypertrophy, meanwhile, PAC and TG were associated with microalbuminuria in PA patients. No factors were found to be related to cerebrovascular damage. Furthermore, multinomial logistic regression analysis also showed that the occurrence of cardiac hypertrophy in PA patients was independently associated with obesity. PAC and TG contributed to the microalbuminuria.

### 3.3. Effect of Lateralization of Aldosterone Secretion on Metabolic Disorders and Total Organ Damage in Patients with PA

We also determined whether lateralization of aldosterone secretion affected total organ damage and metabolic disorders. Patients with PA were divided into bilateral and unilateral hypersecretion groups according to their AVS results. Overall, bilateral PA patients were older than unilateral patients (Supplemental Table S1). As shown in Supplemental Table S2, the incidence of MS (41.1% vs. 46.9%, *P* = 0.28) and IGT/DM (31.9% vs. 42.8%, *P* = 0.07) had no statistically significant difference between unilateral PA and bilateral PA. However, unilateral PA patients had a higher incidence of microalbuminuria (46.0% vs. 29.5%, *P* < 0.01). Furthermore, patients with bilateral PA with MS had significantly increased IVST compared with those without MS. Meanwhile, in the unilateral PA group, LVDd, IVST, LVMI, IMT, Cr, and incidence of cardiac disease were obviously higher among patients with MS than in non-MS patients (shown in Table 4).

## 4. Discussion

In this retrospective study, we demonstrated that MS was a main risk factor for multiple organ complications in patients with PA. First, more severe organ damage was seen in patients with PA with MS. Second, the incidence of MS showed no significant difference in patients with bilateral PA and with unilateral PA, whereas in unilateral PA, the effect of metabolic disorder on organ damage was more severe. In addition, microalbuminuria was higher in patients with unilateral PA. Furthermore, this study found that obesity contributes to cardiac hypertrophy, meanwhile, PAC and TG contribute to microalbuminuria in PA patients. Our study highlights the crucial role of MS components in contributing to the development of organ damage, especially in unilateral PA.

The prevalence of MS and its components has been debated in patients with PA compared to patients with EH, although a number of studies have demonstrated an increased prevalence of insulin resistance, diabetes, hyperglycemia, obesity, and MS [4, 9, 25]. PA is considered the most common curable endocrine hypertension and is typically caused by APA and IHA [26]. Currently, many studies have also focused on the incidence of diabetes and MS in patients with different clinical subtypes of PA. A previous observation suggested that the occurrence of metabolic abnormalities was significantly higher in IHA than in APA, but the prevalence of diabetes did not differ between the two groups [15]. Ohno et al. [12] also showed that the prevalence of diabetes and dyslipidemia did not significantly differ between IHA and APA patients, but IHA patients tended to be obese. However, another recent multicenter cohort study claimed that prediabetes was significantly more prevalent in patients with bilateral hypersecretion of PA than in those with unilateral PA, which was associated mainly with subclinical hypercortisolism [16]. Cortisol excess is associated with metabolic disorders leading to increased cardiovascular risk [27]. Our study found the same trend (IGT/DM: 42.8% vs. 31.9%, *P* = 0.07), but the difference was not statistically significant, likely due to the small number of subjects. However, few studies have further discussed the effect of metabolic disorders on target organ damage in patients with different subtypes of PA.

In recent decades, a series of animal and clinical studies have demonstrated that aldosterone is an independent risk factor for causing renal damage and cardio-cerebrovascular diseases [8, 28]. In animal models, aldosterone has deleterious effects involving cardiovascular disease aside from its effect on electrolyte and blood pressure regulation [29, 30]. Similar findings have been demonstrated in clinical trials. In a 7-year follow-up study, patients who either underwent surgical treatment or were given spironolactone showed a significant decrease in LVM, which was correlated with aldosterone concentration [31]. In addition, the incidence of renal damage in clinical trials has shown great variability [32–35]. Recently, some studies have shown the detrimental effects of aldosterone on metabolic disorders, the mechanism of which caused by reduction of insulin secretion and function [36]. These alterations could contribute to metabolic disorders and type 2 DM in patients with PA. These findings are also in agreement with a meta-analysis showing an increased frequency of abnormal glucose metabolism (higher glucose levels and insulin resistance and lower insulin sensitivity) and rate of cardio-cerebrovascular events in PA compared with EH [6, 37–39]. Adipose tissue can secrete factors that stimulate aldosterone production, and hence, a bidirectional relationship might exist between aldosterone production and visceral obesity [40–42]. In this study, we also showed that obesity was associated with cardiac hypertrophy. These results indicate that excessive aldosterone secretion is associated with the development of MS and that its components contribute to TOD.

However, most studies have not further explored organ damage in PA patients complicated with MS. The differential clinical prevalence of metabolic disorders and related organ damage is unclear. In this study, we focused on the effect of metabolic disorder on organ damage in patients with unilateral and bilateral PA. We confirmed that MS is associated with heart and renal damage in patients with PA, but it was inconsistent with aldosterone elevation. In addition, the effect of MS on organ damage was more remarkable in patients with unilateral PA than in those with bilateral PA.

In conclusion, our current study demonstrated that PA patients with MS may experience more serious target organ damage, and the contribution of MS to the severity of organ damage differs across clinical subtypes. The limitation of this study is the relatively small sample size in a single center and the lack of long-term follow-up data on cardio-cerebrovascular diseases. These should be observed in future prospective studies.

## Figures and Tables

**Table 1:** The clinical and biochemical baseline of the PA patients with or without MS.

Characteristic	Without MS (*n* = 148)	With MS (*n* = 113)	*P* value
Sex (male, %)	30.40	58.40	<0.001^∗∗∗^
Age (y)	47 ± 11.2	52 ± 10.2	<0.001^∗∗∗^
BMI (kg/m^2^)	23.8 (22.3-25.7)	26.5 (25-29.7)	<0.001^∗∗∗^
WC (cm)	80.04 ± 7.98	91.15 ± 7.62	<0.001^∗∗∗^
SBP (mmHg)	131 (144-157)	146 (133-156)	0.39
DBP (mmHg)	90 ± 13.28	87 ± 13.50	0.09
Total cholesterol (mmol/L)	4.30 ± 0.81	4.31 ± 1.12	0.95
Triglycerides (mmol/L)	1.11 (0.81-1.44)	2.01 (1.48-2.57)	<0.001^∗∗∗^
HDL (mmol/L)	1.29 ± 0.27	1.09 ± 0.26	<0.001^∗∗∗^
LDL (mmol/L)	2.74 ± 0.63	2.79 ± 0.80	0.55
IGT/DM (*n*, %)	14 (9.4)	78 (69.0)	<0.001^∗∗∗^
Uric acid (*μ*mol/L)	286 (235.15-334.75)	338.55 (289.12-388.55)	<0.001^∗∗∗^
PAC (ng/dl)	20.1 (16.0-25.0)	19.6 (16.0-23.0)	0.19
PRA (ng/ml/h)	0.50 (0.1-1.0)	0.52 (0.12-1.1)	0.87
Serum potassium (mmol/L)	3.55 ± 0.53	3.51 ± 0.45	0.51
Antihypertensive drugs (*n*)	1.66 ± 0.82	1.81 ± 1.00	0.15

Data are expressed as the mean ± SD, median (25th–75th percentiles), or raw numbers. ^∗^*P* < 0.05, ^∗∗^*P* < 0.01, and ^∗∗∗^*P* < 0.001. PA: primary aldosteronism; MS: metabolic syndrome; BMI: body mass index; SBP: systolic blood pressure; DBP: diastolic blood pressure; HDL: high-density lipoprotein cholesterol; LDL: low-density lipoprotein cholesterol; DM: diabetes mellitus; IGT: impaired glucose tolerance; PAC: plasma aldosterone concentration; PRA: plasma renin activity; WC: waist circumstance.

**Table 2:** Target organ damage in PA patients with or without MS.

	Without MS (*n* = 148)	With MS (*n* = 113)	*P* value
Parameters			
LAD (mm)	31.86 ± 3.46	33.90 ± 3.78	<0.001^∗∗∗^
LVDd (mm)	42.76 ± 3.61	44.17 ± 3.93	0.026^∗^
IVST (mm)	10.64 ± 1.66	11.64 ± 1.66	<0.001^∗∗∗^
LVPW (mm)	10.08 ± 1.48	10.63 ± 1.49	0.012^∗^
IMT (mm)	0.77 ± 0.18	0.85 ± 0.19	0.015^∗^
Creatinine	56.8 (49.5-67.0)	67.3 (56.2-78.4)	0.018^∗∗^
eGFR (ml/min/1.73m^2^)	138.2 (119.8-163.0)	129.0 (111.2-150.1)	0.012^∗^
UACR (mg/g)	20.0 (8.6-57.0)	28.3 (12.6-82.4)	0.05
Incidence of organ damage			
Hypertensive heart disease (%)	29.7	53.1	<0.001^∗∗∗^
Coronary heart disease (%)	7.4	12.3	0.17
Cerebrovascular disease (%)	22.9	44.2	<0.001^∗∗∗^
Chronic kidney disease (%)	37.1	43.3	0.31

Data are expressed as the mean ± SD, median (25th–75th percentiles), or raw numbers. ^∗^*P* < 0.05, ^∗∗^*P* < 0.01, and ^∗∗∗^*P* < 0.001. PA: primary aldosteronism; MS: metabolic syndrome; LAD: left atrial anterior and posterior diameters; LVDd: left ventricular diameters; IVST: interventricular septal thickness; LVPW: left ventricular posterior wall thickness; IMT: intima-media thickness; UACR: urinary albumin to creatinine ratio.

**Table 3:** Multinomial logistic regression analysis for the variable with organ damage.

Variables	Hypertensive heart disease			Microalbuminuria		
	Odds ratio	CI	*P* value	Odds ratio	CI	*P* value
PAC	0.994	0.945-1.045	0.802	1.002	1.001-1.0004	0.010^∗^
Abdominal obesity	1.108	1.066-1.150	<0.001^∗^	N/A	N/A	N/A
Triglyceride	1.082	0.829-1.412	0.563	1.358	1.017-1.813	0.038^∗^

A *P* value < 0.05 was considered statistically significant; PAC: plasma aldosterone concentration; N/A: not applicable.

**Table 4:** Target organ damage in patients with bilateral and unilateral patients with or without MS.

	Bilateral PA			Unilateral PA		
	MS (-) (*n* = 52)	MS (+) (*n* = 46)	*P* value	MS (-) (*n* = 96)	MS (+) (*n* = 67)	*P* value
Parameters						
LVDd (mm)	43.12 ± 3.59	43.7 ± 3.54	0.50	42.55 ± 3.63	44.18 ± 4.18	0.02^∗^
IVST (mm)	10.82 ± 1.77	11.64 ± 1.69	0.04^∗^	10.54 ± 1.60	11.64 ± 1.65	<0.001^∗∗∗^
LVMI	111.2 ± 32.20	112.4 ± 25.81	0.82	104.7 ± 23.40	114.2 ± 24.75	0.04^∗^
IMT (mm)	0.81 ± 0.16	0.80 ± 0.14	0.85	0.76 ± 0.17	0.88 ± 0.21	0.002^∗∗^
Creatinine	67.0 (50.2-69.2)	69.6 (52.1-75.2)	0.73	57.0 (47.5-66.2)	65.7 (57.1-82.7)	0.002^∗∗^
Incidence of organ damage						
Hypertensive heart disease (%)	30.70	50.0	0.05	30.2	56.7	<0.001^∗∗∗^
Coronary heart disease (%)	11.5	6.5	0.39	5.2	16.4	0.01^∗^

Data are expressed as the mean ± SD, median (25th–75th percentiles), or raw numbers. ^∗^*P* < 0.05, ^∗∗^*P* < 0.01, and ^∗∗∗^*P* < 0.001. PA: primary aldosteronism; MS: metabolic syndrome; LVDd: left ventricular diameter; IVST: interventricular septal thickness; LVMI: left ventricular mass index; IMT: intima-media thickness.

## Data Availability

The data that support the findings of this study are available from the corresponding author upon reasonable request.

## Supplementary Materials

Supplementary Materials Supplemental Table 1: the clinical and biochemical baseline of the bilateral and unilateral PA patients. Supplemental Table 2: target organ damage in patients with bilateral PA and unilateral PA.
