# Supplementary material for: Clinical Characteristics of Target Organ Damage in Primary Aldosteronism with or without Metabolic Syndrome
Source: J Diabetes Res. 2022 Sep 7;2022:8932133. doi: 10.1155/2022/8932133 (PMC9473883; doi:10.1155/2022/8932133)
Supplement: Supplementary Materials — Supplemental Table 1: the clinical and biochemical baseline of the bilateral and unilateral PA patients. Supplemental Table 2: target organ damage in patients with bilateral PA and unilateral PA. [file 8932133.f1.docx]

**Supplemental Table 1. The clinical and biochemical baseline of the bilateral and unilateral PA patients.**

| Characteristic | Bilateral PA  (n=98) | Unilateral PA  (n=163) | *P* Value |
| --- | --- | --- | --- |
| Sex (male, %) | 44 | 67 | 0.54 |
| Age(y) | 51 ±9.8 | 48±11.5 | 0.03* |
| BMI（kg/m^2^） | 25.15±3.19 | 25.29±3.27 | 0.73 |
| WC (cm) | 84.85±9.53 | 85.29±9.63 | 0.74 |
| SBP (mm Hg) | 146.57±20.55 | 145.06±20.72 | 0.56 |
| DBP (mm Hg) | 88.85±13.61 | 88.79±13.35 | 0.96 |
| Total cholesterol (mmol/L) | 4.30±0.96 | 4.31±0.95 | 0.95 |
| Triglycerides (mmol/L) | 1.71±1.11 | 1.65±1.13 | 0.62 |
| HDL-c (mmol/L) | 1.18±0.31 | 1.17±0.29 | 0.69 |
| LDL-c (mmol/L) | 2.74±0.66 | 2.78±0.73 | 0.67 |
| Uric acid(umol/L) | 311.97±72.58 | 315.24±91.21 | 0.76 |
| PAC (ng/dl) | 19.9(16.0-24.0) | 19.8(16.4-23.8) | 0.88 |
| PRA (ng/ml/h) | 0.53(0.17-1.07) | 0.32(0.10-0.89) | 0.86 |
| Serum potassium(mmol/L) | 3.59±0.47 | 3.51±0.51 | 0.22 |

Data are expressed as the mean ± SD, median (25^th^–75^th^ percentiles) or raw numbers. * *P* < 0.05. *PA*, primary aldosteronism; *MS*, metabolic syndrome; *BMI*, body mass index; *SBP*, systolic blood pressure; *DBP*, diastolic blood pressure; *HDL*, high-density lipoprotein cholesterol; *LDL*, low-density lipoprotein cholesterol; *DM*, diabetes mellitus; *IGT*, impaired glucose tolerance; *PAC*, plasma aldosterone concentration; *PRA*, plasma renin activity; *WC*, waist circumstance.

**Supplemental Table 2. Target organ damage in patients with bilateral PA and unilateral PA.**

|  | Bilateral PA  (n=98) | Unilateral PA  (n=163) | *P* Value |
| --- | --- | --- | --- |
| MS (n, %) | 46(46.9%) | 67(41.1%) | 0.35 |
| IGT/DM (n, %) | 42(42.8%) | 52(31.9%) | 0.07 |
| **Parameters** |  |  |  |
| LAD（mm） | 33.09±3.3 | 32.54±3.90 | 0.32 |
| IVST（mm） | 11.18±1.77 | 11.01±1.72 | 0.51 |
| IMT（mm） | 0.81±0.16 | 0.81±0.20 | 0.79 |
| Creatinine | 62.4（50.5-73.6） | 59.9（50.0-72.1） | 0.56 |
| **Incidence** |  |  |  |
| Hypertensive heart disease (%) | 39.7 | 41.1 | 0.83 |
| Coronary heart disease (%) | 9.1 | 9.8 | 0.86 |
| Cerebrovascular disease (%) | 29.5 | 35.5 | 0.32 |
| Microalbuminuria (%) | 29.5 | 46.0 | 0.008** |

Data are expressed as the mean ± SD, median (25^th^–75^th^ percentiles) or raw numbers. ** *P* < 0.01. *DM*, diabetes mellitus; *IGT*, impaired glucose tolerance; *LAD,* left atrial anterior and posterior diameters, *LVDd*, left ventricular diameters; *IVST*, interventricular septal thickness; *LVPW*, left ventricular posterior wall thickness; *IMT*, intima-media thickness; *UACR*, urinary albumin to creatinine ratio.
